# King George and Queen Mary

**Published:** 1918-07-13

**Authors:** 


### The Hospital

The Workers' Newspaper of Administrative Medicine and Institutional Life, Administration, National Insurance and Health.

No. 1675, Vol. LXIV. SATURDAY, JULY 13, 1918. PRICE TWOPENCE

### KING GEORGE AND QUEEN MARY.

War is a cormorant of the people's time, energies, and thoughts. It creates such kaleidoscopic changes without notice and without intermission as to compel all who have minds and the power of thought to have it eternally before them, often to their discomfort and not infrequently to the prostration of their strength and energies. The higher the position the greater the weight of care and responsibility due to the war. Yet on the eve of the fifth year of war the nation, for a brief space, has swept it all aside that it might give place to a fitting tribute to their Sovereign and His Consort from the people of all classes throughout His Majesty's Dominions and the nations of our Allies. If we pause for a moment to recall the historical grandeur of the English monarchy, going back to Alfred the Great through the period of the Saxon monarchy to the Norman Conquest, and then on again through Plantagenet, Tudor, and Stuart kings to the present Royal House, we cannot fail to realise something of this grandeur, of its traditions, and the main causes of England's greatness and weight in the councils of the nations of the world to-day.

It is a remarkable fact that more than 130 years have passed since a king and queen of England could have celebrated the twenty-fifth anniversary of their wedding, and that Saturday, July 6, 1918, was the first occasion on which such a celebration has taken place in this country. Their Majesties' Silver Wedding will doubtless be a landmark in British history, and we of to-day can thank God and take courage that, despite the war, everything connected with it was worthy of the occasion, gave heartfelt satisfaction to the whole nation and Empire and to our Allies, and demonstrated the happiness and joy which it brought to their Majesties, their children, and every one of their family, including Her Majesty Queen Alexandra. A beautiful day, producing a great outpouring of the people's love and affection, sympathy and cordiality, in an atmosphere of united rejoicing and great content. Their Majesties spent it in an ideal English home, surrounded by their children, united in their affection to their parents and to each other, the only absent member being H.R.H. the Prince of Wales on duty at the seat of war, a fact which brought the Royal Family, if possible, into closer communion with British families everywhere throughout the Empire.

Forty-one years ago King George became a cadet in the Royal Navy, and since that day his life has been one of continuous activity, including an amount of genuine hard work and hard study and reading which few probably realise. Those who want to know the facts, to qualify to give sound judgment, to become masters in knowledge, to learn how to control others as well as themselves, must likewise make it their business to learn at first hand and to master subjects so thoroughly as to make them capable of offering opinions which necessarily carry weight. Many people will remember the famous speech which King George, then Prince of Wales, made at the Mansion House on his return with the Princess from their visit to the larger part of the wide dominions of which he is the monarch. That speech created widespread interest and carried conviction and weight. It was written by Himself when he resided at York House, St. James', and his call, "Wake up, England," caused a stir which has borne good fruit, and has had results which have no doubt contributed and will continue to contribute to the awakening of the people, and the wonderful position now occupied and to be occupied in the world by the Old Country and the Dominions. In October 1905 His Majesty, then Prince of Wales, with the Princess, paid a visit to India, where he took part in a tiger hunt at Gwalior, and after their return, on the completion of their journey through the Empire, and having so identified themselves with the interests, lives, tastes, and occupations of myriad races, speaking many and diverse tongues, as Lord Curzon declared, they have become the State's greatest public servants, and it is their service, even more than their royal station, that has been their claim to the devotion of the Empire.

Our King is no longer called upon to lead his hosts to battle, but he is a very visible factor in the business and organisation of war. King George's work has been done throughout in such a quiet and unassuming way that he has so far received much less appreciation and credit than he deserves. There are few accessible places on the war fronts, either by land or sea, which His Majesty has not visited in person, stimulating his sailors and soldiers by his presence, applauding and rewarding their valour, condoling with their sufferings, and commiserating with their bereavements. Her Majesty the Queen has been indefatigable in good works, and, as all England knows, the wounded in the hospitals, the nurses in the wards, the workers of many classes and degrees behind the lines, have been cheered and consoled by the gracious presence and kindly words of Queen Mary and of the King himself.

The King's personal work has been thoroughly well done, and his knowledge to-day of the conditions under which his subjects live and work, are governed and provided for, is more complete and thorough, probably, than that possessed by any previous king. Mr. Lloyd George, as Premier, and Mr. Asquith, as leader of the Opposition, bore testimony in the House of Commons to His Majesty's services as statesman, his undaunted courage under the most dismaying conditions, and the unwearying tact and assiduity with which His Majesty strove for peace in July 1914. Before the war their Majesties had more than their share of troubled and anxious times, and our political leaders testify that the King, with the ever-ready sympathy and co-operation of Her Majesty, never lost head, or heart, or nerve, always leant towards policies of reconciliation and appeasement, diligently thought out day by day the problems, whether of his own duty or of the nation's need, showed unfailing consideration for those who had the privilege to serve him, and when he had accepted the final counsels of his constitutional advisers adopted and acted on them with whole-hearted sympathy. England and the Empire have many blessings to be thankful for, and in the circumstances of the present time they have had few greater than the presence on the throne of England to-day of King George and Queen Mary.

